# Causal relationship between health insurance and overall health status of children: Insights from Pakistan

**DOI:** 10.3389/fpubh.2022.934007

**Published:** 2022-12-07

**Authors:** Noshaba Aziz, Tinghua Liu, Shaoxiong Yang, Wioletta Zukiewicz-Sobczak

**Affiliations:** ^1^School of Economics, Shandong University of Technology, Zibo, China; ^2^College of Economics and Management, Northwest A&F University, Xianyang, China; ^3^Department of Food and Nutrition, Calisia University, Kalisz, Poland

**Keywords:** health insurance, child health, propensity score matching, MICS, Pakistan

## Abstract

Evaluating the impact of health insurance always remains a methodologically challenging endeavor due to the absence of sample randomization. This paper evaluates the impact of health insurance on the health status of children in Pakistan using the data of the Multiple Indicator Cluster Survey (MICS) for Punjab, Pakistan, from 2017 to 2018. The study adopted the propensity score matching (PSM) method to address the sample selection bias. The sample is matched on potential covariates such as mother characteristics (education level), household head characteristics (gender, age, and education), and other household conditions (such as home dwelling, internet access, wealth index, migration member, number of children residing in the home, as child illness, etc.). The findings revealed that children with insurance have considerably better health than non-insured, at a 1% significance level. The results confirm that health insurance is not a luxury but a need that improves children's overall health. In this regard, governments should enhance and expand programs related to health insurance, especially for children. Health insurance programs will not only help poor people but also improve the overall infrastructure of health services in the country.

## Introduction

Healthcare is regarded as a fundamental right and critical need of residents of any country (1). The general wellbeing of people and development of countries depend significantly on access to essential healthcare services. Access to healthcare services is still severely constrained in developing nations where poverty and inequality negatively impact health status and impede the delivery of it and care accessibility. Recently, developing countries have also focused on implementing non-profit health insurance programs that provide access to necessary medical care without people experiencing financial distress (2, 3). This medical care covers the full range of primary healthcare services, from wellness promotion to palliative care and everything in between. Numerous nations globally have universal health insurance policies in place. To advance health facilities, the World Health Organization (WHO) also emphasized bolstering healthcare financing reforms (4).

To increase parity in the delivery of healthcare, an increasing number of low- and middle-income nations have chosen healthcare coverage in recent years (5). The fundamental objective was ensuring everyone could get inexpensive, high-quality healthcare services (6–8). This reduces economic obstacles and delivers financial security to marginalized households for healthcare services and medical treatment (2). However, the stress of healthcare costs means that 50% of the world's population still lack access to the necessary healthcare, and 100 million people still fall into poverty every year (9).

Investment in healthcare services for children rather than for older people is regarded as more effective because the better health of children leads to the overall development of society. Moreover, childhood health significantly influences educational and labor outcomes at later ages (10–13). Globally, it has been found that one in every three children experiences malnutrition. The condition is particularly found among the poor, who do not have access to health, education, water, and sanitation. Based on this, health insurance is regarded as a viable option for improving the health status of children to some extent, as previous studies also observed a positive association between health insurance and child health (14–17). In developing countries, more than 70% of children are diagnosed with undernutrition, especially in Asia, and more than 90% of children with stunted growth live in Asia and Africa. Worldwide, children with stunted growth below 5 years of age mainly belong to low-income countries (18).

Additionally, Pakistan was highlighted in this study. It is one of the low-income South Asian nations, with a population of roughly 220 million. Hepatitis B and C are pervasive in Pakistan, where 7.6% of the population has one of the two diseases. It is ranked as having the fifth-highest TB burden globally and has a particularly deadly endemic malaria region. Pakistan has one of the lowest immunization rates among developed nations, with a 60% total vaccination rate. Additionally, even though polio has been eradicated from the rest of the globe, Pakistan is still considered to have an endemic of the illness. Gastroenteritis and respiratory ailments continue to be the leading causes of death in young children. For children younger than the age of five, the frequency of different malnutrition disorders is estimated as follows: underweight (31.6%), wasting (10.5%), severe wasting (3.3%), stunting (45.0%), and overweight (4.8%) (19). Among these, child stunting is highly prevalent in Pakistan, and according to the recent report of the Multiple Indicator Cluster Survey (MICS) (20), stunting (34.3%) is higher in rural areas compared to urban areas (26%). Stunting is a growth abnormality caused by a long-term lack of essential nutrients and the recurrence of diseases (18). Stunting reduces cognitive and physical development, resulting in less working efficiency and poor economic growth and development (21).

Healthcare in Pakistan is currently ranked 154th out of 195 countries in terms of overall system performance. Pakistan struggles to maintain a suitable healthcare system in terms of quality and affordability as a developing country, with just 2% of its GDP allocated to total health expenditures (22). The sporadic service delivery and poor performance accountability within the government continue to be problems for the health system and affects both efficiency and quality. The public sector needs more employees, and working environment and job satisfaction need some improvements. Inadequate resource allocation across various levels of healthcare, as well as an imbalance in the size, skill levels, and deployment of the health workforce, are other issues that the whole health sector must deal with. Even though Pakistan is one of the regions with the lowest health insurance coverage worldwide, the National Health Vision 2016–2025 aims to give a responsive national direction to face the numerous health concerns. The country's health insurance coverage is far below the ideal level, and poverty and illiteracy are the most significant causes of this lack of information about health insurance.

According to the MICS survey, 3.2% of women and 3.9% of males between the ages of 15 and 49 reported having health insurance. Similarly, 3.2% of children between the ages of 5 and 17, and 2.3% of children under the age of five were reported in MICS as having health insurance. However, Pakistan has made broad efforts to offer adequate health facilities. Nevertheless, healthcare service delivery in the community is plagued by several other administrative challenges and resource shortages (23). People are also very cognizant of the healthcare circumstances and the rising costs, and are reluctant to buy health insurance. Due to ignorance and poverty, the majority of Pakistan's population have never felt the need to get health insurance, according to research by Tappis et al. (24). In addition, many believe that rather than paying a deductible for years, they would cover medical expenditures as they arise, particularly in the case of children, because it is believed that young people can never be affected by severe illnesses at a young age. But disease can indeed strike anyone at any time, whether they are young or old; consequently, having health insurance is essential for everyone. In other words, families bear the majority of the costs of healthcare.

In the prevailing literature, many studies explored this phenomenon, but in the case of Pakistan, the research is still sparse. Although some studies highlighted the socio-economic disparities behind health outcomes in Pakistan (25–30), there is an unexpected shortage of empirical studies in the case of health insurance. This study can provide a baseline scenario for health insurance enrolment in Pakistan and help policymakers and planners design health insurance coverage expansion strategies for Pakistan overall. Keeping in mind the importance of health insurance, especially for children who are the future of any nation, it is imperious to conduct research exploring the relationship between health insurance and child health. To meet this study's objectives, the survey data source from MICS has been used to scrutinize the impact of health insurance on child health status by using health proxies such as height-for-age among children below 5 years. This study's findings should provide insights to policymakers to strengthen and enhance health insurance policies to sustainably overcome poor health in children and accomplish universal health insurance coverage, especially in Pakistan. From a broader view, this paper generally enhances the health insurance and child health literature in developing countries, especially in Pakistan.

The remaining sections of the study are as follows. The covariates on which health-insured and non-insured children are matched are explained in “Covariates of health insurance” section. In “Methods” section, the data sources and data strategy are presented. The empirical findings in the form of results are described in “Results” section. The results are discussed in “Discussion” section. Finally, the study concludes with policy implications and mentions future research limitations.

## Covariates of health insurance

Health insurance in Pakistan is linked to several factors, so this section explicitly discusses the covariates which influence health insurance. The health insurance scheme's primary goal is to protect low-income families against financial risk and improve their health status by providing better healthcare services. According to standard economic theory, health insurance minimizes health expenses for impoverished people (16). The beneficial effects of health insurance are highly recognized in many developed countries (17); however, in the case of developing countries the results are inconsistent, where Indonesia (31), Philippines (32), and India (33) showed less response to even extremely subsidized insurance schemes. Likewise, an initiative in 2007 was conducted by Karnataka, an Indian state, to provide free in-patient care to individuals falling below the poverty line. The evaluation report by Rajasekhar et al. (34) revealed that even after 2 years, the enrolment rate in the program was only about 68%, and utilization of the services also remained low. After enrolment to the program, only 0.4% of the beneficiaries received treatment within 6 months.

It is assumed that health insurance is influenced by other specific attributes of the families in which the individuals are born. It has been argued that many socio-economic disparities are accountable for accessing childhood healthcare services (25). For instance, the education of the population (35), especially the mother's level of education (23), is crucial. Many researchers posited that education is vital in providing more efficient health inputs to children (25–27). The research reflects that educated mothers may pay for the optimum preventative care by visiting doctors, thereby more effectually averting serious diseases (28). In contrast, uneducated mothers cannot seek better and more adequate healthcare services or care due to a lack of knowledge and capability. So in this vein, it is likely that availing of health insurance benefits could be affected by mothers' educational levels. Several other studies also demonstrated a significant impact of parental education in vaccination coverage (36, 37).

Likewise, variables of household head such as gender, age, and educational attainment are also incorporated, as they may affect the households' general environment and influence the obtainment of child health insurance. Household conditions, such as home dwelling and wealth score, are also added. According to Geberselassie et al. (29), children with highly stunted growth belong to poor households. They are very susceptible to impaired physical growth because of frequent contagions and a lack of appropriate care. In this case, it is unlikely that poor households will opt for health insurance schemes due to already existing financial constraints. Hence, it is probable that household conditions play an essential role in health insurance coverage decisions. People from wealthy strata have less mental stress and are willing to acquire better health supports (30, 38). Moreover, according to Meng et al. (39), internet exposure influences decisions to avail of health insurance coverage. Further, in the case of households with a member who has migrated and works elsewhere, having increased financial support by way of receiving remittances also favors health insurance uptake. In addition, families of a child with an illness are also inclined toward purchasing health insurance. Sick individuals are more inclined to avail of health-related benefits, and not including these individuals in the analysis may lead to biased results.

The number of children under the age of five and between 15 and 17 years is also added to the model to address the probability that the connection between age and the decision to avail of child insurance is related. Large families may wish to lower the amount they pay for health insurance, particularly in deprived households. The reason is mainly due to financial constraints that influence a family's choice to fulfill other basic needs. Moreover, individuals eligible for such a health insurance program mostly live in rural settings and must travel to the district card distribution center. However, children are not required to pay a yearly premium to become insured, they just need to register at the local district office. Therefore, geographical proximity to hospitals, traveling costs, and forgone daily earnings may deter obtaining health insurance. Therefore, based on the above discussion, it has been found that all variables mentioned are essential for controlling channels of obtaining health insurance, and so they have been included in this study.

## Methods

### Data sources

The Islamic Republic of Pakistan encompasses four provinces, named Punjab, Baluchistan, Sindh, and Khyber Pakhtunkhwa, of which Punjab is by far the largest and most densely populated. This study used sub-national level data from the MICS for Punjab, Pakistan, for 2017–2018. This is an extensive household survey established by UNICEF; it mainly focuses on issues concerning children and women covering all 36 districts of Punjab. In 2,692 sample clusters, the overall sample was 53,840 households. The MICS also includes information on health insurance membership and anthropometric measurements for children. Complete data of 38,047 children less than 5 years old were selected for the empirical analysis of this study. This study also refers the reader to the Punjab Bureau of Statistics Planning & Development Department document for further methodological facts.

### Outcome variable

The outcome of interest is the child's health status. For exploring the child's health status, a proxy variable such as height-for-age (HAZ) *z*-score is used, as it is a frequently used measure to assess the child's health status. According to Thomas et al. (40), children's weight usually fluctuates short term and reflects their current health status, while height, on the other hand, reflects the long-term health of the child. Height assesses linear growth that can be affected by long-term nutrition deficiency and frequently occurring chronic diseases. Therefore we use HAZ as a proxy to measure the child's health (41).

### Explanatory variable

**Figure 1:**
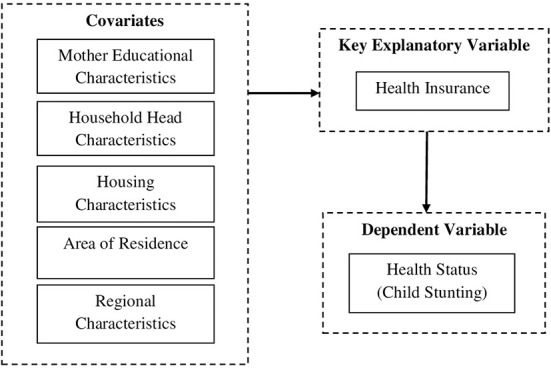
Framework operationalized in the current study.

Figure 1 depict the operational framework of the study. The key explanatory variable in the study is health insurance. A dummy variable is created to define health insurance and is equal to one if the child is covered by any health insurance and zero if the child has no insurance.

### Covariates

Comparing the health of children having insurance to that of children having no insurance is not likely to reflect the effects that having insurance has on health. Health insurance is expected to be connected to other specific health determinants. Therefore, it is advisable to use the covariates to match the control group with the same units parallel to the treatment group (42). Here, this involves coupling non-insured children with insured children who are analogous to each other but only differ in insurance status. As an initial estimation, each household with child insurance in our sample is matched with demographically similar families with no child insurance. Then, a range of individual, household head, and economic conditions are added (as discussed in “Covariates of health insurance” section). Moreover, all insured and non-insured children's information is retrieved from the same data source and drawn from an identical population.

### Empirical estimation

The main methodological problem in health-insurance-related studies is sample selection bias. Therefore, this study employed propensity score matching (PSM) to remove the concern that similar aspects could drive health insurance and health outcomes. According to Nichols (14), conventional regression is employed for assessing causal inference of effects when confounding variables are directly measured. However, this can lead to selection biases when the covariate correlates with the residual (i.e., endogenous) (14, 15). If selection bias arises only because of observed characteristics, and the model includes all observed confounders likely to influence child health insurance and child health, then PSM would be a more appropriate tool to use. Based on non-experimental data, PSM is extensively used to assess the treatment's impact (43). It considers the propensity score (probability) for each treated and untreated individual and matches individuals from the treated group with one or multiple individuals from the untreated group that have identical propensity scores. With this approach, the numerous observable differences are reduced to a difference of one dimension, i.e., a treatment-induced difference (44). The primary objective of the PSM is to assess the average treatment effect related to health insurance, based on the outcome of the average treatment effect in the treated (ATT). To explore the phenomenon empirically, the current research employs the following steps:

The fitted value (the value of propensity score) of the conditional probability of children having insurance is estimated by the logistic model


(1) $$\begin{array}{l} PS_{m}=Pr\;\left[L_{m}=1|X_{m}\right]=E\;\left[L_{m}=0|X_{m}\right] \end{array}$$

where *L*_*m*_ = 1 refers to children who have health insurance, and *L*_*m*_ = 0 indicates children who do not have health insurance. *X*_*m*_ signifies the observable covariates, such as mother, household head, and household economic conditions. The treatment and the control group are matched based on possible covariates. To enhance the reliability of the conclusions, the k nearest neighbor matching method is employed, which is based on matching individuals by looking for k individuals from different groups with the closest propensity score. In this study, *k* is set to *1*; in other words, one-to-one matching is executed to lessen the mean square error.

*ATT* is used to compute the health status difference between the treatment and the control group. Finally, the health insurance's impact on the child's health is attained.


$$\begin{array}{l} ATT=E\left(D_{1m}|L_{m}=1\right)-E\left(D_{0m}|L_{m}=1\right)\\ \;=E\left(D_{1m}-D_{0m}|L_{m}=1\right) \end{array}$$

*D*_1*m*_ is the health status of children having health insurance, *D*_0*m*_ is the health status of children not having health insurance, *E(D*_1*m*_*|L*_*m*_ = 1*)* can be directly observed, and *E(D*_0*m*_*|L*_*m*_ = 1*)* cannot be directly observed and is a counterfactual outcome. Consequently, PSM is a suitable approach for creating the corresponding substitute index.

### Patient and public involvement statement

Study participants or the public were not involved in the design, conduct, reporting, or dissemination plans of our research.

## Results

### Impact of covariates on child health insurance by logistic regression

Health-insured children are likely to receive healthcare in time, and they are less prone to being hospitalized than non-insured children. However, multiple barriers may inhibit access to health insurance. Knowledge and attitudes of families also play an essential role in deciding where to seek proper care for children, therefore child insurance was primarily assessed by using the multivariate logistic model based on the following confounders: mother's educational level, household head characteristics, household conditions, and place of residence and region. The results are reported in Table 1. The confounders have been discussed in detail in the previous sections.

**Table 1:** Estimation of covariates on health insurance (logistic regression).

**Covariates**	**Co. eff**	**Odd. ratios**	**Std. err**	**Marginal effect**	**Std. err**
**Mother educational characteristics**					
Illiterate	−0.788^***^	0.455^***^	0.144	−0.017^***^	0.144
Primary education	−0.455^***^	0.635^***^	0.123	−0.009^***^	0.122
Middle education	−0.164	0.849	0.123	−0.164	0.123
Higher education	−0.172^***^	0.842^*^	0.103	−0.172^***^	0.103
Secondary education	Omitted	Omitted			
**Household head characteristics**					
Gender	0.204	1.226	0.137	0.004^***^	0.003
Age	0.014^***^	1.014^***^	0.003	0.000^***^	0.000
No education	−1.049^***^	0.349^***^	0.12	−0.023^***^	0.003
Primary education	−1.070^***^	0.343^***^	0.129	−0.023^***^	0.003
Middle education	−0.775^***^	0.460^***^	0.122	−0.017^***^	0.003
Secondary education	−0.479^***^	0.619^***^	0.097	−0.010^***^	0.003
Higher education	Omitted	Omitted			
**Housing characteristics**					
House dwelling	0.747^***^	1.078^***^	0.023	0.002^***^	0.001
Internet	0.015	1.015	0.073	0	0.002
Remittance	−0.773^***^	0.462^***^	0.134	−0.017^***^	0.003
Child illness	−0.102	0.902	0.071	−0.002	0.002
Wealth index scores	0.324^***^	1.383^***^	0.058	0.007^***^	0.001
No. of children aged <5	−0.097^***^	0.908^***^	0.031	−0.002^***^	0.001
No. of children aged 15–17	0.028	1.029	0.018	0.001	0.004
**Area of residence**					
Rural area	−0.200^***^	0.818^***^	0.083	−0.004^***^	0.002
**Regional characteristics**					
Bahawalpur	−2.247^***^	0.106^***^	0.348	−0.049^***^	0.008
Dera Ghazi Khan	−1.377^***^	0.25243^***^	0.246	−0.030^***^	0.005
Faisalabad	−0.652^***^	0.521^***^	0.147	−0.014^***^	0.003
Gujranwala	−1.074^***^	0.342^***^	0.15	−0.023^***^	0.003
Lahore	−0.649^***^	0.523^***^	0.149	−0.014^***^	0.003
Multan	−0.687^***^	0.503^***^	0.159	−0.149^***^	0.004
Rawalpindi	0.896^***^	2.450^***^	0.122	0.019^***^	0.003
Sahiwal	−1.137^***^	0.321^***^	0.226	−0.025^***^	0.005
Sargodha	Omitted	Omitted			
-Cons		0.067^***^	0.021		
Pseudo *R*^2^	0.154				
Wald chi^2^	1593.86				
Log Pseudo likelihood	−3611.512				
Observations	38,047				

*, *** Represent the significance level of 10 and 1%, respectively.

### Common support domain

**Figure 2:**
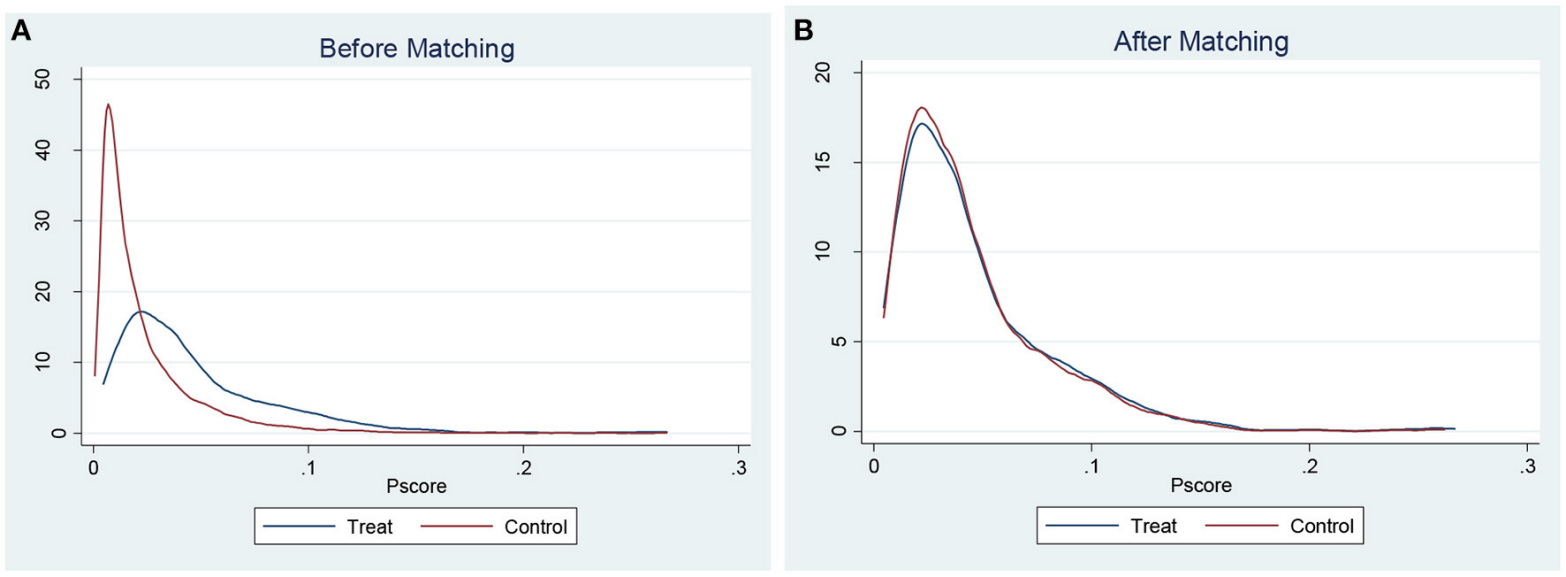
**(A)** Propensity score distribution before matching. **(B)** Propensity score distribution after matching.

The first step in PSM is to identify common support areas that reflect overlap in the values between the treatment group and control groups. The area of common support for the treatment group (Treat) and the control group (Control) is shown in Figures 2A,B to confirm matching quality. The graphs of function density before and after PSM are also presented in these figures. The propensity score values of children having and not having insurance generally overlap. Thus, it is believed that the data used in this study have better conditions for the common support domain, and maximum observations are within the range of common value.

In addition, Table 2 displays the maximum loss of sample size, and according to the findings, the sample loss is slight. The results show that the treatment and the control group lost 3,526 and 4 samples, respectively, with the remaining 34,517 being the total of matched samples.

**Table 2:** Sample matching results.

**Result of sample matching**	**Unmatched sample**	**Matched sample**	**Total**
Untreated	3,526	33,618	37,144
Treated	4	899	903
Total	3,530	34,517	38,047

Source: Author's estimates.

### Balance test

The next step is to perform a balance test. This test assesses the quality of matching by comparing significant covariates between the treatment group and control groups. The analysis eliminates individuals from the treatment group whose propensity score is outside the range of the propensity scores of the control group (i.e., who are not in the common support area). The balancing properties' overall matching quality indicators are presented in Table 3. The results show that the standard deviation between the two groups after matching is reduced from 24.4 to 4.1. The overall bias is significantly reduced, indicating that the matching quality is sufficient. The likelihood ratio shows that the joint significance test of covariates is highly significant at the 1% level; nevertheless, the test value is no longer significant after matching. In addition, the pseudo-*R*^2^ value is reduced from 0.158 before matching to 0.014 after matching. In conclusion, these results show that there is no significant difference between children with health insurance and those without insurance. The test results reveal that the current study's matching method effectively balances the distribution of the covariates between the control and treatment groups and successfully minimizes sample selection bias.

**Table 3:** Test for matching method quality.

**Sample**	**Unmatched**	**Nearest neighbor matching**
Ps *R*^2^	0.158	0.014
LR Chi^2^	1345.86	33.72
*P* > Chi^2^	0.000	0.142
Mean bias	24.4	4.1
Med bias	19.3	2.6

Source: Authors' estimates.

### Covariates balance results between treated and untreated groups before and after matching

For all covariates, the summary statistics for matched and unmatched groups are presented in Table 4. To compare the treatment groups, the group means in the first two columns and standardized percentage bias, percentage bias reduction, t-statistics, and significance are presented in columns three to six. The first row of each covariate in Table 2 compares the covariate means between treated and control groups for the unmatched sample. The covariates significantly differ between both groups. For example, the treated group has a higher amount of members than the control group. The same is found in the context of household head characteristics, where the level of education was higher in the treated group than the control group. The same difference is found for other covariates, as shown in the table below. A significant difference for all covariates in both treated and control groups would be problematic when assessing the effect of treatment. The balance and differences of mean statistics show that the control and the treated groups are balanced for most of the covariates (second row for each covariate), representing no systematic differences between the groups after matching (45). The standardized difference for most covariates is highly significant at 1%. Generally, the findings of balance for all covariates suggest the suitability of assessing the ATT by comparing the two groups.

**Table 4:** Covariate balance between treated and control groups before and after matching.

**Summary statistics**	**Unmatched/** **matched**	**Mean treated**	**Mean control**	**% Bias**	**% Reduction bias**	***t*-test**	***P*-value**
**Mother's educational characteristics**							
No education	Unmatched	0.174	0.443	−60.9		−16.17	0.000
	Matched	0.175	0.152	5	91.7	1.28	0.202
Primary education	Unmatched	0.169	0.204	−8.8		−2.53	0.011
	Matched	0.170	0.207	−9.4	−7.1	−1.99	0.047
Middle education	Unmatched	0.135	0.103	10.1		3.18	0.001
	Matched	0.136	0.142	−2.1	79.5	−0.41	0.683
Secondary education	Unmatched	0.215	0.129	22.6		7.46	0.000
	Matched	0.216	0.222	−1.8	92.1	−0.34	0.732
Higher education	Unmatched	0.307	0.121	46.6		16.76	0.000
	Matched	0.304	0.276	7	85.1	1.3	0.194
**Household head's characteristics**							
Gender (male = 1)	Unmatched	1.081	1.075	2.3		0.69	0.488
	Matched	1.080	1.089	−3.3	−44.5	−0.68	0.498
Age	Unmatched	49.218	46.360	19.3		5.72	0.000
	Matched	49.170	49.549	−2.6	86.7	−0.54	0.586
No education	Unmatched	0.199	0.395	−43.9		−11.95	0.000
	Matched	0.200	0.197	0.7	98.3	0.18	0.859
Primary education	Unmatched	0.114	0.189	−21.2		−5.76	0.000
	Matched	0.115	0.107	2.2	89.7	0.53	0.559
Middle education	Unmatched	0.132	0.141	−2.7		−0.79	0.430
	Matched	0.132	0.158	−7.5	−177	−1.54	0.124
Secondary education	Unmatched	0.286	0.179	25.4		8.2	0.000
	Matched	0.287	0.308	−5	80.1	−0.98	0.327
Higher education	Unmatched	0.269	0.095	46.4		17.44	0.000
	Matched	0.266	0.230	9.5	79.6	1.75	0.081
**Housing characteristics**							
House dwelling (yes = 1)	Unmatched	1.684	1.601	5.3		1.6	0.109
	Matched	1.681	1.716	−2.2	58.5	−0.46	0.648
Wealth score	Unmatched	0.604	−0.075	78.6		20.46	0.000
	Matched	0.597	0.601	−0.4	99.5	−0.1	0.918
Household having migrant member (yes = 1)	Unmatched	0.897	0.106	−5.4		−1.54	0.123
	Matched	0.090	0.085	1.9	65.1	0.42	0.676
Child illness (yes = 1)	Unmatched	1.712	1.732	−4		−1.18	0.237
	Matched	1.713	1.667	9	−125.9	1.99	0.047
No. of children aged <5	Unmatched	1.999	2.186	−17.2		−4.81	0.000
	Matched	1.997	1.934	5.5	67.9	1.25	0.212
No. of children aged 15–17	Unmatched	1.794	2.038	−13.5		−3.67	0.000
	Matched	1.799	1.805	−0.4	97.3	−0.08	0.932
**Area of residence**							
Rural (yes = 1)	Unmatched	1.351	1.261	19.7		6.1	0.000
	Matched	1.352	1.343	2.2	88.9	0.45	0.656
**Regional characteristics**							
Bahawalpur	Unmatched	0.009	0.091	−37.6		−8.44	0.000
	Matched	0.010	0.007	1.6	95.9	0.78	0.437
Dera Ghazi Khan	Unmatched	0.023	0.112	−35.9		−8.44	0.000
	Matched	0.023	0.018	2.2	93.7	0.83	0.406
Faisalabad	Unmatched	0.097	0.123	−8.2		−2.34	0.019
	Matched	0.098	0.093	1.4	82.8	0.32	0.749
Gujranwala	Unmatched	0.097	0.172	−22		−5.9	0.000
	Matched	0.098	0.079	5.6	74.7	1.41	0.158
Lahore	Unmatched	0.113	0.124	−3.4		−1	0.318
	Matched	0.113	0.113	0	100	0	1.000
Multan	Unmatched	0.076	0.118	−14.2		−3.88	0.000
	Matched	0.077	0.099	−7.5	47.1	−1.67	0.096
Rawalpindi	Unmatched	0.838	0.095	84.1		33.94	0.000
	Matched	0.436	0.408	6.8	91.9	1.19	0.233
Sahiwal	Unmatched	0.028	0.066	−18.3		−4.63	0.000
	Matched	0.028	0.034	−2.6	85.6	−0.68	0.494
Sargodha	Unmatched	0.116	0.098	6.1		1.87	0.061
	Matched	0.117	0.149	−10.4	−71.9	−2.02	0.044

Source: Authors' estimations.

### Treatment effect estimation

Finally, the treatment effect (health insurance) on the outcome variable (child health) is assessed and reported in Table 5. The ATT measured by the nearest neighbor method shows a significant effect at a 1% significance level. It is a positive number, indicating that insurance significantly improves the child's health after amending the selection bias.

**Table 5:** Effect of health insurance on child stunting.

**Sample**	**Unmatched**	**Average treatment effect on treated (ATT)**
Treated	0.2081	0.2091
Control	0.3177	0.1598
Difference	−0.1095	0.0493^***^
Standard error	0.0156	0.0201
T-statistics	−7.000	2.44

*** Represents the significance level at 1%.

## Discussion

The main aims of this study were to show the effects of having health insurance on child health in Pakistan by using nationally representative data. Children with health insurance are more likely to receive early healthcare services than those not insured. Multiple barriers may affect access to health insurance. Hence, the current study used propensity score matching. In the existing literature, various researchers posited that the uptake of health insurance is affected by several factors such as gender, education, wealth, family size, area of residence, household structure, ethnicity, and religion (46, 47). Therefore, before moving to propensity score matching and exploring the average treatment effect in the treated, this study primarily employed a logistic model to estimate the impact of different covariates—maternal educational level, household head characteristics, household conditions, place of residence, and region—that are likely to influence insurance and health outcomes, as shown in Table 1.

The assessment is of intrinsic interest as it reveals astonishing findings. Previous research posited that educated women are more aware of the advantageous effects of health insurance uptake and its importance for the better health of their children. Previous studies found that child mortality falls when women become more educated (28). Similarly, in the case of vaccine uptake, several studies pointed to a positive correlation with mothers' education (26, 48–50). However, our results revealed that increased education decreases uptake of health insurance, which infers that education in Pakistan does not play the biggest role, and that lack of health facilities and infrastructure, especially if they are mismanaged and misgoverned, can affect decision-making. Therefore, education alone cannot support health insurance uptake. Moreover, Pakistan is a dominant male society, and social and cultural barriers limit women's decisions regarding health insurance for children. In this case, education cannot help women to obtain health insurance for children. Furthermore, it infers that educated mothers prefer access to alternative treatment sources.

Looking at household characteristics, it was found that household age significantly impacts the probability of insurance, as it may affect the household's general environment. For instance, household heads with increased age are well aware of health insurance benefits and the likelihood of improving child health. Moreover, in the context of housing, for children from a wealthier background, having a house dwelling increases the chances of obtaining health insurance. A better wealth position lessens the mental stress of considering financial outlay, and people are willing to acquire health insurance. The results align with the study of Hajizadeh (38) and Raza et al. (51), where it was revealed that wealth plays a crucial role in improved healthcare. On the other hand, children with family members who have migrated and siblings below 5 years have a lower probability of being insured. Migration is an expensive effort and needs investment initially, and is thus likely to influence a family's decision to purchase health insurance. As migration requires an initial investment, families belonging to low economic strata have financial constraints and may cut the health insurance expenditure from their budget.

Moreover, having health insurance is found to be negatively significant for all regions except Sahiwal, where the results are positive. There are differences between regions regarding infrastructure and development, such as health facilities and roads, which are also likely to influence uptake of health insurance and healthcare facilities. Many people suitable for the insurance programs live in rural areas and must travel long distances to the district's card distribution office. Though children are not required to pay an annual premium to get insurance, they must register at the adjacent district office. Therefore, geographical proximity to hospitals and transportation costs hinder uptake of health insurance. The regions in Pakistan also have different vaccination coverage, as revealed by Asif and Akbar (27). On the other hand, gender of household members, internet access, and child illness do not considerably influence insurance status, nor does having children below 15 years of age.

In the context of health insurance, our results elucidate the significant positive effect on child health after adjusting the observable covariates of insured and uninsured children, as discussed above. After matching, the health insurance effects on a child's health are found to be positively significant in Pakistan. Undeniably, health insurance is advantageous as it reduces the financial constraints related to medical expenses and offers accessibility to care that otherwise would be expensive to afford (52). Furthermore, it may allow individuals to access proper healthcare, which otherwise may be sacrificed or delayed by them due to budget constraints (53). Similarly, Liu et al. (36) in the case of Rwanda, revealed that community-based health insurance enhanced the utilization of medical services and minimized huge medical expenditure by households. Another advantage of health insurance is that it increases the family's exposure to better health professionals and ultimately results in better health for their child after obtaining better advice from healthcare personnel. The results align with previous studies (54, 55). Currie and Gruber (56) described how the Medicaid entitlement considerably lessened child mortality by 12.77%. Likewise, Howell et al. (57) also showed a 3% decline in child mortality with an increase of 10% in Medicaid entitlement. Furthermore, Joyce and Racine (58) also discovered that the Children's Health Insurance Program enhanced the health status of children. Most studies posited that healthcare services are improved by having health insurance coverage (57, 59).

In particular, children who have Medicaid benefit from boosted education, economic conditions, and health outcomes (60). Having health insurance was found to improve health status in India (2), Mexico (3), Taiwan (4), but not in China (5, 6) or Costa Rica (7). Several other studies also support our findings and revealed that maternal and child health improves by expanding national health insurance (61–63). Specifically, as Nshakira-Rukundo et al. (64) revealed, child stunting declined by 4.3% due to the participation of households in community health insurance programs over 1 year. The results proposed that the extension and development of community health insurance programs could be beneficial in boosting health along with financial security and service utilization in rural settings. It has also been revealed that having children's health insurance improves health and reduces long-term dependency on social safety nets (65). Many other health programs at the community level have also been found to be more effective in reaching out to the majority of the susceptible population by addressing financial, geographical, and socio-cultural obstacles (66). These programs can act as instruments to increase the capacity of marginalized people to pay for medical services. Likewise, the conditional cash transfer scheme also contributed to reducing stunting among children under 5 years old (67). In contrast, Miller and Wherry (8) found that expanding Medicaid under the Affordable Care Act (ACA) did not improve the individual's health status. Likewise, Courtemanche et al. (9), from the Behavioral Risk Factor Surveillance System data, found that ACA did not prove successful in improving respondents' self-reported health status. In addition, several other studies also showed no significant association of health insurance entitlement with children's health outcomes (7).

The existing literature reveals several studies that have used PSM to assess the effects of health insurance uptake. For example, Trujilo et al. (16) discovered that Colombia's impoverished and uninsured people seek medical care more frequently than those in a subsidized insurance program. Similarly, Thuong et al. (68) assessed the effects of updated health insurance law in Vietnam on the use of outpatient and in-patient care services, healthcare service use at various levels of providers, kinds of providers, and types of visits across various entitlement categories. The PSM was used to analyze the data, and it was discovered that having health insurance is the factor that has the most significant impact on medical services at health centers. Nevertheless, the effects of having health insurance on the increase in the frequency of municipal health station visits, the number of visits for groups with health insurance at provincial hospitals, and the number of visits to health facilities for health checks and consultations were sensitive to unobserved characteristics. A similar methodology was employed in a recent study by Nicholson et al. (69), which found that children with intellectual disabilities were no more likely than children without intellectual disabilities to have seen a general practitioner or emergency room in the previous 12 months. The Mother and Child Health Handbook (MCH) was determined by Kawakatsu et al. (70) as a helpful tool for enhancing Kenyans' health literacy and health-seeking behavior. Using PSM, the study found that factors influencing ownership of the MCH Handbook include the child's sex, the caregiver's relationship to the child, maternal age, health knowledge, birth interval, and household wealth index. Mensah et al. (71) applied a similar methodology in the instance of Ghana and evaluated health indicators of women who recently registered in the national health insurance program, with those who are not balancing the essential background characteristics of the respondents. The results showed that health insurance is a valuable tool for enhancing health outcomes for those with it, prompting the Ghanaian government to intensify enrollment promotion, especially among the underprivileged.

This study proposes specific policy implications based on the empirical analysis, such as prioritizing children's health insurance as possibly the most significant and attainable way forward to boost child's health. As per the authors' knowledge, the present study is the first in Pakistan to use data to scrutinize health insurance's role in improving child health status. A relatively large sample size helps us evaluate the actual effect and infer valuable conclusions. The findings of this study, however, are subjected to certain limitations. Firstly, the nature of data, i.e., cross-sectional, limits us from discerning the causal association between the explanatory and outcome variables. Moreover, though health insurance successfully improves insured children's health status in Punjab, Pakistan, gains might not share the same across regions. So the current study sets avenues for future researchers to explore the regional heterogeneity and investigate the mechanism.

## Conclusion

This study examined the impact on child health of having child health insurance using the MICS 2017–18 data for Pakistan. Moreover, the study adopted the propensity score matching method to address sample selection bias. The findings reveal that health insurance is a plausible factor in improving child health. Previous studies have also concluded this and documented that the benefits having health insurance in childhood in linked to substantial health benefits during adolescence and adulthood. Further, it has been proposed that policymakers should find a solution to the obstacles that prevent the advancement of child health. Moreover, local people should be aware of the effectiveness of health insurance and its productive outcome on children's lives as they grow older. At the local level, several factors influence health insurance enrollment. In this regard, electronic media can play an efficient role in creating awareness regarding health insurance. This is possible through mobile applications or automated messages delivering health insurance-related information. Policymakers should identify the difficulties people confront while accessing health insurance. The government should facilitate people to be able to obtain health insurance in their local communities and to further increase health insurance coverage to improve their health status. Moreover, Pakistan has already realized that it is time to move toward the concept of health insurance, both at the governmental and individual levels. The government is currently working to provide health insurance programs for those with financial disadvantages. Realizing that healthcare costs can lead to individuals becoming impoverished, it is essential to ensure that everyone has financial access to healthcare in order to prevent people from being forced into poverty or forgoing treatment they are unable to purchase. In terms of limitations, this study specifically focuses on Pakistan, but it would be interesting to conduct panel analysis to further confirm the impact that having child health insurance may have in developing countries.

## Data availability statement

Publicly available datasets were analyzed in this study. This data can be found at: https://microdata.worldbank.org/index.php/catalog/3559.

## Author contributions

NA performed the data analysis and wrote the first draft of the manuscript. TL did analysis and review the manuscript. SY and WZ-S critically revised the manuscript and made essential corrections. All authors contributed to the article and approved the submitted version.
